# Inclusion complexation of the anticancer drug pomalidomide with cyclodextrins: fast dissolution and improved solubility

**DOI:** 10.1016/j.heliyon.2021.e07581

**Published:** 2021-07-15

**Authors:** Zoltán-István Szabó, György Orbán, Enikő Borbás, Dóra Csicsák, Szabina Kádár, Béla Fiser, Máté Dobó, Péter Horváth, Eszter Kiss, Lívia Budai, Judit Dobos, Tamás Pálla, László Őrfi, Gergely Völgyi, Gergő Tóth

**Affiliations:** a Department of Pharmaceutical Industry and Management, George Emil Palade University of Medicine, Pharmacy, Science and Technology of Targu Mures, Gh. Marinescu 38, RO-540139, Targu Mures, Romania; b Department of Pharmaceutical Chemistry, Semmelweis University, Hőgyes E. u. 9, Budapest H-1092, Hungary; c Department of Organic Chemistry and Technology, Budapest University of Technology and Economics, Műegyetem rakpart 3., Budapest, H-1111, Hungary; d Computational Molecular Design Research Group, Institute of Chemistry, Faculty of Materials Science and Engineering, University of Miskolc, H-3515, Egyetemváros-Miskolc, Hungary; e Ferenc Rákóczi II. Transcarpathian Hungarian Institute, Beregszász, Transcarpathia, Ukraine; f Department of Pharmaceutics, Semmelweis University, Hőgyes Endre u. 7, Budapest, H-1092 Hungary; g Vichem Chemie Research Ltd, Hermann O. utca 15, H-1022, Budapest, Hungary

**Keywords:** Pomalidomide, Cyclodextrin complexation, Inclusion complex, Solubility, Comparative dissolution, Pomalyst®

## Abstract

Pomalidomide (POM), a potent anticancer thalidomide analogue was characterized in terms of cyclodextrin complexation to improve its aqueous solubility and maintain its anti-angiogenic activity. The most promising cyclodextrin derivatives were selected by phase-solubility studies. From the investigated nine cyclodextrins – differing in cavity size, nature of substituents, degree of substitution and charge – the highest solubility increase was observed with sulfobutylether-β-cyclodextrin (SBE-β-CD). The inclusion complexation between POM and SBE-β-CD was further characterized with a wide variety of state-of-the-art analytical techniques, such as nuclear magnetic resonance spectroscopy (NMR), infrared spectroscopy (IR), circular dichroism spectroscopy, fluorescence spectroscopy as well as X-ray powder diffraction method (XRD). Job plot titration by NMR and the A_L_-type phase-solubility diagram indicated 1:1 stoichiometry in a liquid state. Complementary analytical methods were employed for the determination of the stability constant of the complex; the advantages and disadvantages of the different approaches are also discussed. Inclusion complex formation was also assessed by molecular modelling study. Solid state complexation in a 1:1 M ratio was carried out by lyophilization and investigated by IR and XRD. The complex exhibited fast-dissolution with immediate release of POM, when compared to the pure drug at acidic and neutral pH. Kinetic analysis of POM release from lyophilized complex shows that Korsmeyer-Peppas and Weibull model described the best the dissolution kinetics. The cytotoxicity of the complex was tested against the LP-1 human myeloma cell line which revealed that supramolecular interactions did not significantly affect the anti-cancer activity of the drug. Overall, our results suggest that the inclusion complexation of POM with SBE-β-CD could be a promising approach for developing more effective POM formulations with increased solubility.

## 1 Introduction

**Figure 1:**
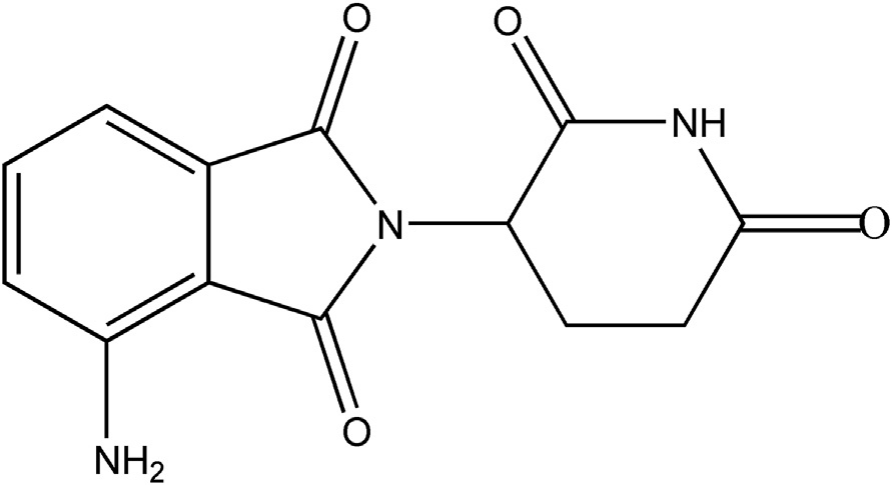
Chemical structure of pomalidomide.

Multiple myeloma is a cancer of the white blood cells, characterized by an increase of abnormal plasma cells in the bone marrow and monoclonal proteins in the serum, often with osteolytic bone lesions. The thalidomide analogue immunomodulatory drugs are first-line treatment options of multiple myeloma in combination with bortezomib and dexamethasone [1]. One of the newer members of the immunomodulatory class of drugs is pomalidomide (POM). POM ((*RS*)-4-Amino-2-(2,6-dioxopiperidin-3-yl)isoindole-1,3-dione, Figure 1) was approved in 2013, by the FDA and EMA as a treatment for relapsed and refractory multiple myeloma. It has been approved for use in patients who have received at least two prior therapies including lenalidomide and bortezomib and have demonstrated disease progression on or within 60 days of completion of the last therapy [2].

POM has a low oral bioavailability mainly due to its poor solubility in water. In rats and monkeys, the oral bioavailability was in the range of 13%–15% following a 100 mg/kg dose. However, the bioavailability in monkeys was high (approximately 100%) at a lower dose of 2 mg/kg, indicating that the extent of absorption is solubility limited [3]. Increasing the solubility of POM could enhance its bioavailability. Consequently, the dose and side effects could be reduced, and possibilities of novel parenteral formulations would also arise.

Solubility enhancement can be achieved by various methods, such as solid dispersion, size reduction, cosolvency, polymorphism, formation of water-soluble prodrugs or complexes [4]. Cyclodextrin (CD) complexation is one of the most extensively investigated methods to improve drug solubility and concomitantly, also bioavailability [5].

The conical structure of CDs and the outside orientation of the hydroxyl groups confer unique physicochemical properties to these cyclic sugars, capable of being solubilized in an aqueous medium and at the same time encapsulating hydrophobic molecules in their internal cavity space. CD complexation of pharmaceuticals can result in improved properties of the guest molecules, such as improved solubility, stability, masking of undesirable properties, protection against oxidation, light-induced reactions, and loss by evaporation [6]. The native CDs contain six, seven or eight *D*-glucose units and are named α-, β- and γ-CD, respectively. Nowadays hundreds of semi-synthetic derivatives of native CDs are available in the market, which extend the range of applications of these molecules [7].

In our recent work CDs were used as chiral selectors to separate POM enantiomers in capillary electrophoresis and high-performance liquid chromatography [8, 9]. In the latter case the β-CD-based chiral stationary phase was applied, and the inclusion complex was characterized to shed light on the underlying mechanisms of enantiodiscrimination [9].

To the best our knowledge, to date, no previous method regarding solubility improvement of POM has been described. The solubility of lenalidomide, another commercially available thalidomide analog, was improved via cocrystal formation with urea and 3,5-dihydroxybenzoic acid [10]. The most studied structurally related drug is the parent molecule, thalidomide. Earlier works prove that CD complexation can improve the solubility and stability of thalidomide [11, 12]. Moreover, thalidomide administered orally in combination with sulfobutylether-β-CD (SBE-β-CD), led to a significant delay in tumor progression because of improved cellular drug absorption and distribution through solubilization in mice [13].

The present work focuses on the characterization of complex formation between POM and various CDs focusing mostly on solubility enhancement and dissolution profile. Subsequently, the *in vitro* anti-proliferative effects were also investigated to verify the effect of the CD complexation on cytotoxicity. The proposed information could offer a molecular basis for a novel, improved drug formulation.

## 2 Materials and methods

### 2.1 Materials

POM was obtained from Beijing Mesochem Technology Co. Ltd. (Beijing, China). D_2_O, DMSO-*d*_6_ and DMSO were ordered from Sigma-Aldrich Hungary (Budapest, Hungary). All native CDs (α-, β- and γ-CD) and their derivatives (hydroxypropyl-β-CD (HP-β-CD) with different degrees of substitution (DS) 3, 4.5, 6; randomly methylated-β-CD DS~12 (RAMEB), permethylated-β-CD (TRIMEB), sulfobutylether-β-CD DS~7 (SBE-β-CD)) were the products of Cyclolab R&D Ltd. (Budapest, Hungary). All other chemicals used were of analytical grade from commercial suppliers. Ultrapure, deionized water was prepared by a Milli-Q Direct 8 Millipore system (Milford, MA, USA).

### 2.2 Physico-chemical characterization

The acid dissociation constant of POM was determined by ^1^H NMR titration using dichloroacetic acid, sarcosine, and sodium acetate as *in situ* pH indicators [14]. The ionic strength was 1M. The *n*-octanol/water partition coefficient (*P*) was determined by the classical stir-flask method based on our earlier published methods [15] at three different *n-*octanol/water phase ratios (0.5, 1, and 2). The partitioning was performed at 25 °C and pH 7. The value of thermodynamic solubility was measured as indicated in phase-solubility methods.

### 2.3 Phase-solubility methods

Phase-solubility analysis was performed according to the method described by Higuchi and Connors [16]. Excess amount of POM (10 mg) was added to 3 mL water (without any buffer) containing increasing concentrations of CDs (0, 5, 10, 15, 20, 25, 30 mM for each CDs, except β-CD, where 0, 2, 4, 8, 10, 12 mM were used, due to its low solubility in water). The obtained suspensions were stirred for 24 h at 25 ± 1 °C. POM concentrations were measured from the filtered suspensions (PVDF 0.22 μm filter membrane) spectrophotometrically on a Jasco V-550 instrument (Jasco Ltd., Tokyo, Japan) at 390 nm. Three parallel measurements were carried out in all cases. The apparent stability constant *K*_1:1_ was calculated from the slope of the initial linear portion of POM concentration against CD concentration profiles assuming 1:1 complex formation in each case (Eq. 1):

(1) $$K_{1:1}=\frac{tg\alpha }{S_{0}\left(1-tg\alpha \right)}$$

Where *tgα* is the slope of phase solubility plot, *S*_o_ is the intrinsic solubility of POM, determined experimentally. Three parallel measurements were performed in each case.

### 2.4 Circular dichroism and fluorescence spectroscopy

Electronic circular dichroism and fluorescence experiments were performed on a Jasco J-810 spectropolarimeter (Jasco Ltd., Tokyo, Japan) in cylindrical cuvettes. POM was dissolved in DMSO to form 0.1 M stock solutions, which were further diluted with distilled water to 0.001 M. The POM concentration was constant during the titration, while the CD concentration increased until 1:30 POM:CD ratio. The spectra were accumulated three times with a bandwidth of 2 nm and a scanning step of 0.1 nm at a scan speed of 100 nm/min. The calculation of the stability constant for the 1:1 M ratio complex had been published previously [17]. The same method has been used for the determination of POM – SBE-β-CD stability constant.

The parallel measured fluorescence intensity can also be used for the calculation of the complex stability constant (*K*), but the own, specific fluorescence of the guest and the complex should also be considered, thus the measured signal is less selective than ellipticity. In this case, the measured signal is the sum of the fluorescent intensity of the guest and the complex. Both the molecule and the complex have a different intrinsic fluorescent activity, *I*_*POM*_ for the guest and *I*_*complex*_ for the complex. The measured fluorescent activity is a linear combination of the two intensities as Eq. (2) described.

(2) $$I=I_{\text{POM}}*{\left[\text{POM}\right]}_{E}+I_{\text{CD}}*\left[\text{complex}\right]$$

where I_POM_ and I_CD_ are the intrinsic fluorescent activity of the guest (POM) and host (SBE-β-CD), respectively; [POM]_E_ is the equilibrium concentration of POM.

Since the equilibrium concentration of POM can be expressed in terms of the total guest and the complex concentrations, Eq. (3) takes on the following form:

(3) $$I=I_{\text{POM}}*{\left[\text{POM}\right]}_{\text{T}}+\left(I_{\text{complex}}-I_{\text{CD}}\right)*\left[\text{complex}\right]$$

*I*_POM_ can be calculated from the pure guest concentration and intensity, while *K* and *I*_complex_ should be calculated with nonlinear parameter estimation as described in our earlier work (Eq. 4) [17]:

(4) $$\left[\text{complex}\right]=\frac{{\left[\text{POM}\right]}_{\text{T}}+{\left[\text{CD}\right]}_{\text{T}}+\frac{1}{K}-\sqrt{{\left({\left[\text{POM}\right]}_{\text{T}}+{\left[\text{CD}\right]}_{\text{T}}+\frac{1}{K}\right)}^{2}-4{\left[\text{POM}\right]}_{\text{T}}\left[\text{CD}\right]}}{2}$$

### 2.5 NMR experiments

All NMR measurements (^1^H NMR titrations, Job plot method) were carried out on an Agilent Varian Unity Inova DDR spectrometer (599.9 MH for ^1^H) (Santa Clara, CA, USA) with a 5 mm inverse detection gradient (IDPFG) probehead at 25 °C. Standard pulse sequences and processing routines available in VnmrJ 2.2 C/Chempack 4.0 were used. Due to the poor solubility of POM, DMSO-*d*_6_ (10 v/v%) was added to the D_2_O solution. During titrations, the POM concentration was kept constant at 1 mM, while CD concentration gradually increased from 0 to 30 mM. For determination of the stability constant Eq. (5) was used:

(5) $$\delta_{obs,}=\delta_{POM}+\text{Δ}\delta \frac{{\left[\text{POM}\right]}_{\text{T}}+{\left[CD\right]}_{T}+\frac{1}{K}-\sqrt{{\left({\left[POM\right]}_{T}+{\left[CD\right]}_{T}+\frac{1}{Κ}\right)}^{2}-4{\left[POM\right]}_{T}{\left[CD\right]}_{T}}}{2{\left[POM\right]}_{T}}$$

where. $\text{Δ}\delta =\delta_{\text{POM}-\text{CD}}-\delta_{POM}$

Stability constants (*K*) of the inclusion complexes were calculated by non-linear parameter fitting of Eq. (4) to the *δ*_*obs*_ versus *[CD]*_*T*_ datasets using OriginPro 8 program based on previous work [18].

### 2.6 Molecular modeling

**Figure 2:**
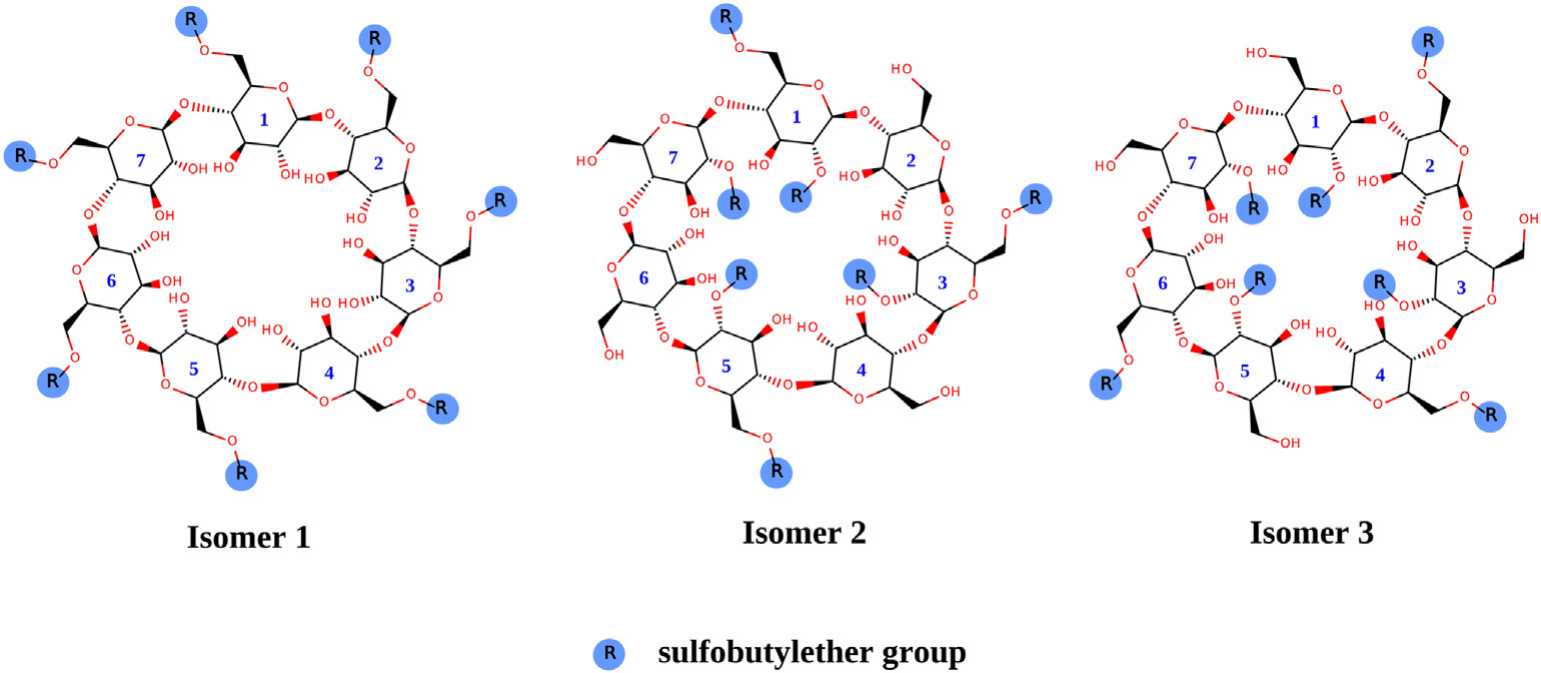
2D structures of SBE-β-CD model isomers.

To explore the interaction of the *S*-enantiomer of POM and SBE-β-CD at the molecular level, molecular modeling tools were applied. The exact SBE-β-CD structure is unknown, because the sulfobutylether (SBE) groups are randomly arranged on the β-CD skeleton and the CD itself is a mixture of derivatives with different degrees of substitution. Therefore, three different isomers were designed based on previous studies to mimic different possible SBE-β-CD structures [19, 20]. It is known that the degree of substitution of SBE-β-CD is ~7 and thus, the model structures carry seven properly arranged SBE groups on the β-cyclodextrin skeleton (Figure 2). The model structures have been prepared (energy minimization, OPLS-AA and Generalized Born implicit solvation model) and used as hosts in molecular docking simulations. By using a “blind docking” protocol [21], the interactions between *S*-POM and the SBE-β-CD isomers have been explored. AutoDock Vina was used to carrying out the docking calculations [22]. The exhaustiveness, the maximum number of binding modes, and the size of the grid box was set to 8, 9, and 30 × 30 × 30 Å, respectively. The most favorable *S*-POM-SBE-β-CD complex from each calculation was selected and analyzed.

### 2.7 Preparation of physical mixture

Physical mixture (PM) was prepared by blending previously sieved (125 mm) POM and SBE-β-CD (1:1 M ratio), in a ceramic mortar.

### 2.8 Preparation of POM - SBE-β-CD inclusion complex by lyophilization

Equimolar amounts of POM (0.1 M) and SBE-β-CD (0.1 M) were dissolved in ethanol-water mixture (1:1 v/v ratio). The mixture was stirred for 8 h at room temperature and filtered through a 0.45 mm Millipore membrane filter. The yellow filtrate was frozen and then lyophilized in a freeze-dryer (ScanVac CoolSafe freeze dryer, Labogene, Denmark) for 24 h. The obtained powders were sieved (125 μm) and kept in a desiccator until use.

### 2.9 Infrared spectroscopy

Infrared spectra (4000–400 cm^−1^) of solid samples were recorded using a Bruker Tensor 37 type Fourier transform infrared (FT-IR) spectrometer (Bruker Corporation, Billerica, MA, USA) equipped with DTGS (deuterated triglycine sulphate) detector with a resolution of 4 cm^−1^. Before testing, the samples were mixed with potassium bromide (KBr) powder and cold-pressed into a suitable disk for FT-IR measurement.

### 2.10 X-ray powder diffraction method

XRD of the samples were recorded by means of a PANalytical (Amelo, the Netherlands) X'pert ProMDP X-ray diffractometer using Cu-Kα radiation (1.542 Å) and a Ni filter. The applied voltage was 40 kV, while the current was 30 mA. The samples were analyzed between 4° and 42° 2ϴ.

### 2.11 Real-time monitoring of dissolution using *in situ* UV probes

The POM concentration (both for the active pharmaceutical ingredient and the complex) versus time (0–30 min) profile was investigated at 25 ± 0.1 °C in Britton-Robinson buffer pH 7.0 and in simulated gastric fluid at pH = 1.6 with UV-probes attached to the Rainbow Dynamic Dissolution Monitor of the μDISS Profiler™ (Pion Inc., Billerica MA, USA). Powder samples, equivalent to 5 mg of pure POM were weighed in 20 mL of appropriate buffer.

### 2.12 Drug release kinetic

To analyze the profile of *in vitro* POM release from LP, the open source KinetDS 3.0 software was used [23] following the method described by Al-Qubaisi et al. [24]. The results of drug release were fitted to kinetic models of zero-order, first-order, second-order, Higuchi, Korsmeyer-Peppas, Weibull, Hixson-Crowell and Michaelis–Menten [25]. The best-fit determines the model that best described the drug release curve, mainly by the coefficient of determination (R^2^), empirical coefficient of determination (R^2^_empirical_) (Eq. 6), Akaike information criterion (AIK) (Eq. 7), Bayesian information criterion (BIC) (Eq. 8) and Root-mean-squared error (RMSE) (Eq. 9).

(6) $$R_{empirical}^{2}=1-\frac{{\sum_{i=1}^{N}\left(y_{i}-{\hat{y}}_{i}\right)}^{2}}{\sum_{i=1}^{N}{\left(y_{i}-y_{AV}\right)}^{2}}$$

(7) $$AIC=2k+N[\ln\left(\sum_{i=1}^{N}{\left(y_{i}-{\hat{y}}_{i}\right)}^{2}]\right)$$

(8) $$BIC=N\;\ln\left(\sum_{i=1}^{N}{\left(y_{i}-{\hat{y}}_{i}\right)}^{2}\right)+k\;\ln\left(N\right)$$

(9) $$RMSE=\sqrt{\frac{\left(\sum_{i=1}^{N}{\left(y_{i}-{\hat{y}}_{i}\right)}^{2}\right)}{N}}$$

where $y_{i}$:observed value, ${\hat{y}}_{i}$: model-predicted value, $y_{AV}$: average output vale.

### 2.13 Proliferation assay

The human myeloma cell line LP-1 was provided by the 1^st^ Department of Pathology and Experimental Cancer Research, Semmelweis University (Budapest, Hungary). Cells were maintained *in vitro* as monolayer cultures in RPMI-1640 medium supplemented with 5% FBS and antibiotic-antimycotic solution at 37 °C in a humidified 5% CO_2_ atmosphere. For the proliferation assay, cells were seeded into 96-well plates at a density of 4000 cells/well containing either POM or the complex, each at various concentrations (10-point dilution series of the compounds were used in the range of 0.6–300 μM). Control cells were incubated in culture media without the active compound. The cells were incubated for 72 h and then a colorimetric assay using MTT was performed to follow cell proliferation. Briefly, 0.5 mg/mL of the dye MTT was added to the wells. After 4 h incubation (37 °C) the medium was gently removed, the plates were air-dried, and the formazan crystals formed in viable cells were dissolved in DMSO. The absorbance at 570 nm was measured with an Analyst GT multimode reader (Molecular Devices, Sunnyvale, CA). The XLfit (IDBS) software was used to generate the dose-effect curves for IC_50_ determination.

## 3 Results and discussion

### 3.1 Physico-chemical characterization of POM

As a preliminary investigation, the acid dissociation constant, the partitioning coefficient, and the intrinsic solubility of POM were determined. The obtained data are summarized in Table 1. From the results, it is notable that there is a second p*K*_a_ for POM with a predicted value of around eleven (the authors found no previous work in the literature describing this parameter based on experimental data). However, due to the poor solubility of the compound in basic media, this parameter was not measurable using ^1^H NMR titration. Moreover, the second p*K*_a_ has not much real practical significance because of the decomposition of POM in basic media. The molecule is in its neutral form in a wide pH range, and it is in its protonated form only in very acidic pH values. The obtained data indicate low solubility and low lipophilicity, which is independent of pH. Our data seems to underline the inclusion of POM as a BCS class IV drug [3, 26]and justified the application of CD complexation to increase POM solubility.

**Table 1:** Results of physico-chemical characterization of POM (mean ± SD, n = 3).

Physico-chemical parameter	Obtained value
Protonation macroconstant (p*K*_a_)	1.89 ± 0.01
Partitioning coefficient (log*P*)	0.01 ± 0.05
Intrinsic solubility (*S*_0_)	4.51 ± 0.11 mg/L

### 3.2 Phase-solubility study

**Figure 3:**
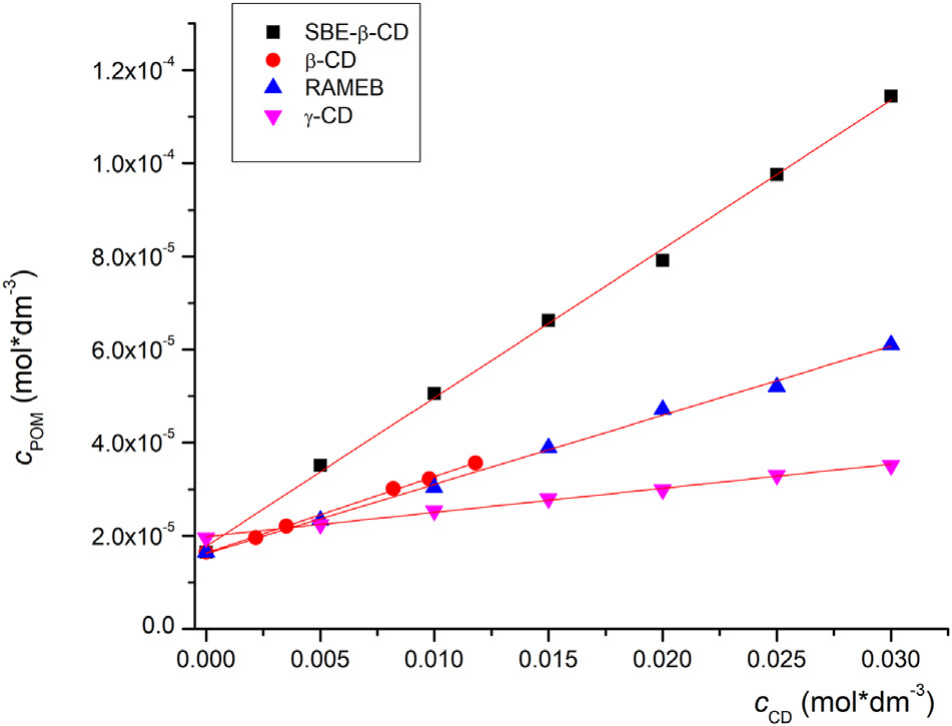
Some representative phase solubility diagrams of POM with different CDs.

Phase-solubility studies are commonly applied to characterize CD complexation in a liquid state. This technique offers valuable information on stoichiometry and complex stability. In the present phase-solubility study, the complexation of POM with nine different CD derivatives was investigated in water. Three native CDs were chosen to select the appropriate cavity size for inclusion of POM. Among synthetic β-CD derivatives the most commonly used ones were selected, which are official in the Pharmacopoeias (HP-β-CD, SBE-β-CD) or approved as excipients in pharmaceutical products intended for human use (RAMEB) [27]. Furthermore, another frequently employed, well-characterized, methylated derivative (TRIMEB) was also studied. In all cases A_L_-type phase solubility diagrams were observed. As the slope of the curve is less than 1 1:1 binding stoichiometry can be assumed. The calculated stability constants based on Eq. (1) and the obtained solubility enhancements are summarized in Table 2, while some representative phase solubility plots are depicted in Figure 3.

**Table 2:** The calculated stability constants and the achieved solubility enhancement based on phase solubility study.

Cyclodextrin	*K* (mean ± SD, n = 3)	Maximum solubility enhancement (CD concentration)
α-	2.7 ± 0.5	1.10 × (30mM)
β-	97.2 ± 3.1	2.16 × (11.8 mM)
γ-	30.3 ± 0.8	1.70 × (30 mM)
HP-β- DS 3	68.5 ± 1.1	3.27 × (30 mM)
HP-β- DS 4.5	129.9 ± 2.2	4.66 × (30 mM)
HP-β- DS 6	87.7 ± 2.8	3.69 × (30 mM)
RAMEB	101.7 ± 1.7	3.75 × (30mM)
TRIMEB	24.0 ± 0.6	1.74 × (30mM)
SBE-β-CD DS 7	194.6 ± 1.0	6.93 × (30mM)

Table 2 shows that the most stable complex with the native CDs is formed with β-CD, indicating that it has the most suitable cavity size for POM, while the complex stability with α-CD is the lowest. Among the tested CD derivatives, only HP-β-CD with DS 4.5 and SBE-β-CD presented higher complex stability with POM than the native β-CD. Interestingly, when comparing the HP-β-CDs with different DSs, the highest stability constant was found with the DS 4.5. Both a decrease and increase in DS lead to a decrease in complex stability. This could be explained by an overall higher strength of interaction with an increase of DS from 3 to 4.5. However, a further increase in the number of substituents per CD molecule might lead to overcrowding and lowers the strength of interactions during inclusion complexation. These results further underline the need to use well-characterized CDs, especially with regard to their DS [28]. Based on our data, methylation of β-CD decreased the complex stability, however this effect is spectacular in the case of TRIMEB. It can be seen that RAMEB forms a three-times more stable complex with POM than TRIMEB. Orgován et al. observed a similar phenomenon when studying clotrimazole CD complexation [29]. Based on our measurements SBE-β-CD was the most suitable host for POM, as it presented the highest complex stability with POM, therefore, this derivative was chosen for further characterization. In a previous study Kale et al. used SBE-β-CD for molecular encapsulation of the parent drug thalidomide with SBE-β-CD to increase its bioavailability [13]. It should be noted that our study is the first to compare the solubility enhancement of one of the thalidomide-derivatives.

### 3.3 NMR Job plot

**Figure 4:**
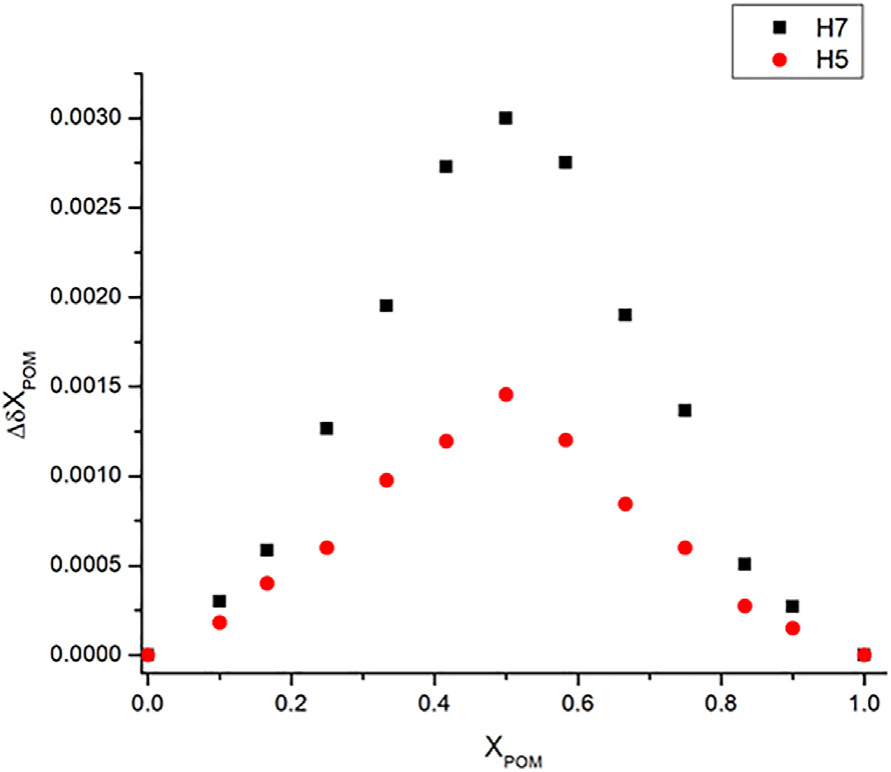
Representative Job plot curves of POM with SBE-β-CD.

Job's continuous variation method was adopted to verify the stoichiometry of the POM-SBE-β-CD inclusion complex. In this experiment, the ^1^H NMR chemical shifts (δ) were measured at different POM concentration/SBE-β-CD concentration ratios, while the sum of c_POM_ + c_SBE-B-CD_ was kept constant. The calculated factors (Δδχ_POM_) were plotted as a function of POM molar ratio (χ_POM_). The resulting Job plot diagrams showed maxima at 0.5 for all investigated protons, indicating 1:1 binding stoichiometry in accordance with phase solubility results. Representative Job plot curves are shown in Figure 4.

### 3.4 Determination of POM-SBE-β-CD stability constant using different spectroscopic techniques

Although the phase-solubility method is one of the easiest and most employed technique for the characterization of inclusion complexes, it should also be noted that the phase solubility plot can only be used to estimate complex stoichiometry, especially for substances with poor solubility [30, 31]. Therefore, alternative techniques such as fluorescence-, circular dichroism-, and NMR spectroscopy, were also applied to determine the stability constant of POM-SBE-β-CD complexes. The measured stability constants were compared with our earlier published data obtained with capillary electrophoresis [8]. The results were summarized in Table 3.

**Table 3:** Comparison of POM-SBE-β-CD stability constant (log*K*) determined by different method (mean ± SD, n = 3).

	Phase solubility	Fluorescence	Circular dichroism	NMR∗∗	CE∗∗∗
1∗	2.29 ± 0.03	2.31 ± 0.01	2.44 ± 0.06	2.08 ± 0.01	2.37 ± 0.01
2∗				2.25 ± 0.01	2.42 ± 0.02

∗ Enantiomer-specific value (First and second enantiomer, respectively).

∗∗ Determined in DMSO/water mixture.

∗∗∗ Literature value [8] pH = 6.5 in 50 mM Tris-acetate buffer.

The stability constants are in good agreement with each other. It can be observed that the complex is characterized by moderate stability sufficient to potentially increase POM solubility. The experiments performed to provide an opportunity to compare the five different techniques. The smallest stability constant was measured by NMR. Due to the low solubility of POM in water, DMSO was applied to reach the appropriate concentration for NMR measurement. DMSO interacts with CDs, which might be one of the reasons for the lowest recorded value [32]. On the other hand, NMR and capillary electrophoresis are also suitable for the determination of enantiospecific complex stability constants. POM is a racemic mixture that exists in two enantiomeric forms. The phase-solubility method, circular dichroism and fluorescence spectroscopy are not capable to distinguish between the enantiomers, the obtained values are averaged for the two enantiomer-CD complexes. The advantage of circular dichroism study in the case of racemic compounds is that the induced circular dichroism signal is directly proportional with complex formation. Fluorescence spectroscopy on the other hand is a simple, fast method and it is the most sensitive one, for the characterization of inclusion complexation.

### 3.5 Molecular modeling study of the POM-SBE-β-CD inclusion complex

**Figure 5:**
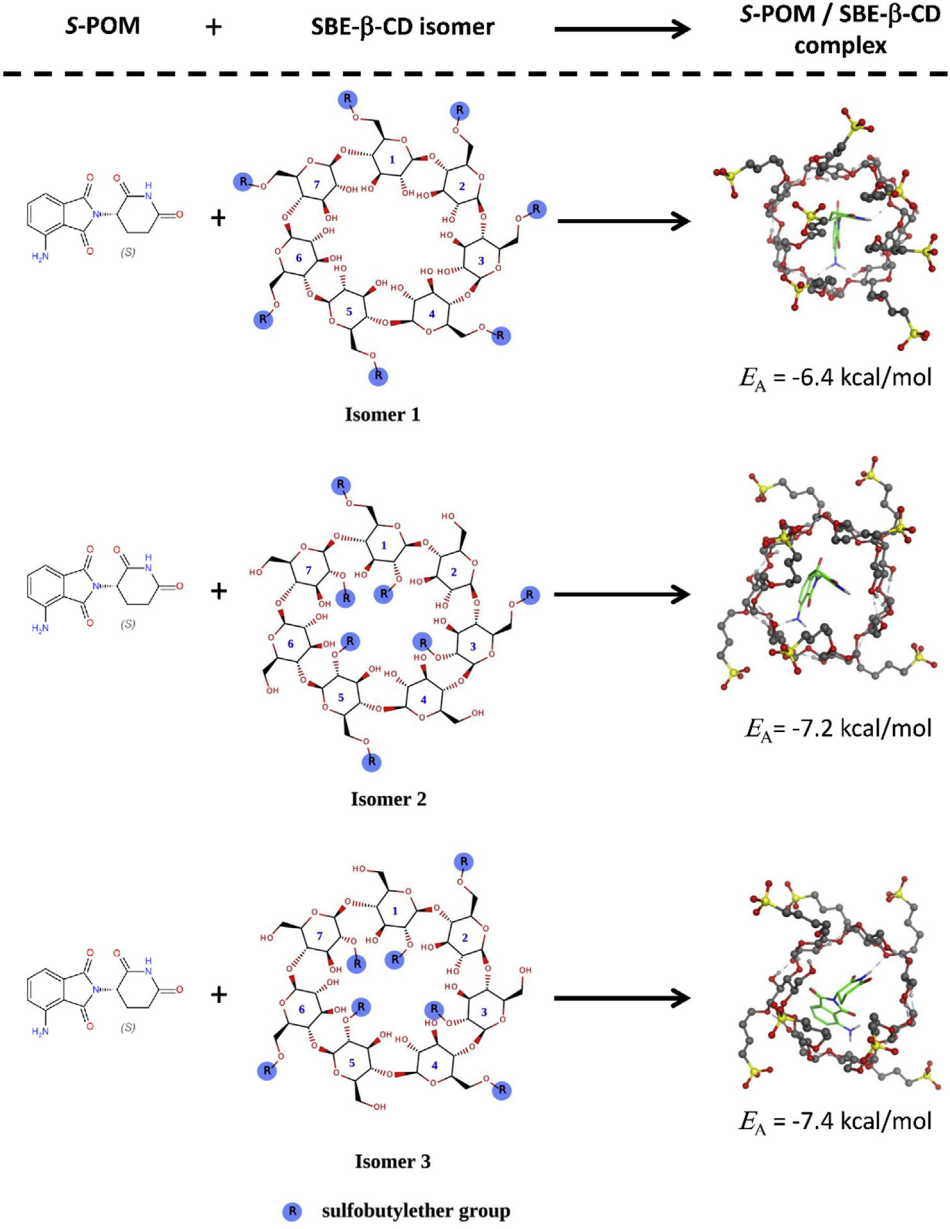
3D structures of *S*-POM-SBE-β-CD complexes with the calculated binding affinities (*E*_A_, kcal/mol).

Docking simulations were used to study the geometric aspects of the complexation of *S*-POM and SBE-β-CD. The 3D structures of the complexes of *S*-POM and three different SBE-β-CD model isomers together with the corresponding *E*_A_ values (binding affinities) can be seen in Figure 5. The binding affinities were between -6.4 to -7.4 kcal/mol, indicating that complex formation is thermodynamically favored regardless of the applied isomer. However, isomer 1 was less favored than the other two SBE-β-CD model structures. Thus, although *S*-POM formed a complex with the SBE-β-CD isomers, the stability values of the complexes are different. To further investigate this phenomenom, single isomer CDs would be necessary. From a structural point of view, it can be observed that the glutarimide moiety of POM is inside the CD cavity in all cases, while the phthalimide part of the molecule interacts with the SBE-β-CD isomers through its primary amine group, which keeps it closer to the CD skeleton.

### 3.6 Solid-state characterization of complex

Apart from the liquid state characterization, solid complexes were also prepared by lyophilization and further analyzed by complementary solid-state techniques.

#### 3.6.1 X-ray powder diffraction

**Figure 6:**
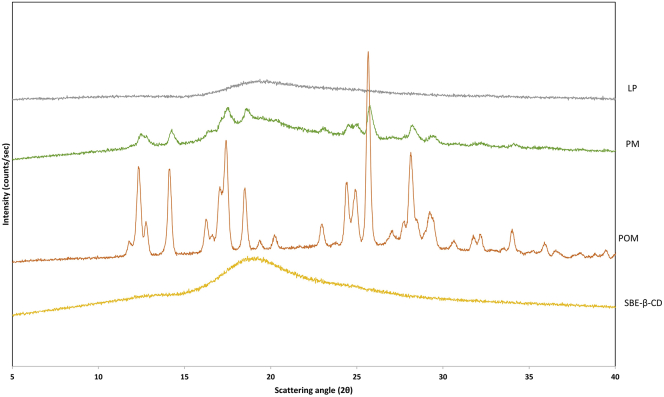
X-ray powder diffraction pattern of POM, SBE-β-CD, PM, and LP.

The results of the XRD analysis are shown in Figure 6. The sharp diffraction peak of POM at 12.4°, 14.1°, 17.4°, 18.5°, 24.5°, 25°, 25.6°, and 28.2° indicates its crystalline nature. SBE-β-CD exhibits a diffuse halo pattern without distinct peaks due to its amorphous nature. The powder diffraction pattern of the PM was basically the superposition of SBE-β-CD and POM diffraction patterns, indicating that simple mixing did not induce complexation of the active. The lower intensity POM peak in the physical mixture is due to the relatively low POM content in the mixture. Simple mixing did not induce interactions between POM and the applied CD. On the other hand, the XRD analysis of LP clearly shows that the inclusion complex is totally in the amorphous state, no sign of crystalline character is present on this diffraction pattern.

#### 3.6.2 FT-IR

**Figure 7:**
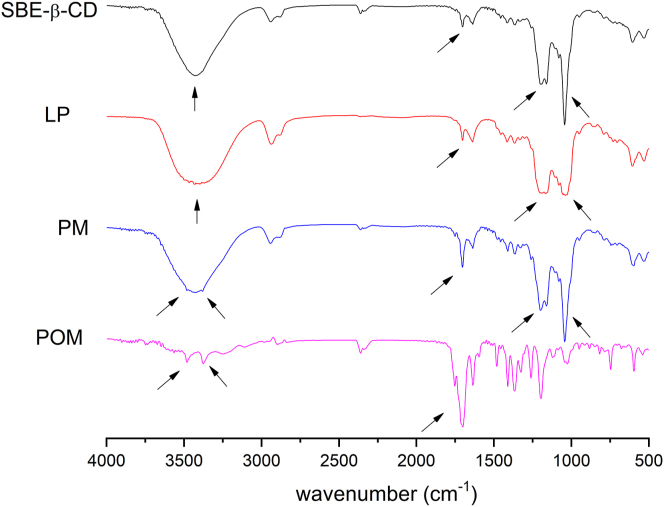
Comparative FT-IR spectra of POM, SBE-β-CD, PM, and LP. Arrows show differences in the obtained FT-IR spectra.

Solid state characterization of POM-SBE-β-CD complexation was undertaken by analyzing the FT-IR spectra of POM, SBE-β-CD, their physical mixture (PM) and the lyophilized product (LP). FT-IR is widely applied for solid state characterization of CD complexes, by tracking the disturbances in vibrational modes through complexation as a window to decipher possible host-guest interactions. Usually disappearing or broadening of peaks, intensity variations or shifts in wavenumber can indicate the complex formation [33]. Figure 7 shows the overlay of the obtained FT–IR spectra.

POM has a complex fingerprint region of the IR spectra due to the presence of heterocycles. The two intense twin bands at 3475 cm^−1^ and 3370 cm^−1^ (aniline N–H stretch), together with the highest intensity peak at 1700 cm^−1^ (C=O stretch) are characteristic for the compound. FT-IR spectra of SBE-β-CD showed a few characteristic absorption peaks, in line with literature data [34], including a strong band at 3423 cm^−1^ (O–H stretching), 2938 cm^−1^ (C–H stretching), 1161 cm^−1^ (C–H vibrations) and 1043 cm^−1^ (C–O stretching). The FT-IR spectra of the PM is a superposition of the spectra of the two individual components. However, when comparing the lyophilized product (LP) with the PM, several differences can be observed. The disappearance of the POM peaks at 1700 cm^−1^ (C=O stretch) and the twin peaks at 3475 cm^−1^ and 3370 cm^−1^ (aniline N–H stretch), alongside with prominent peak broadening both at the O–H stretch (3423 cm^−1^) 1161 cm^−1^ and 1043 cm^−1^ (C–O stretch) of the CD confirm the presence of secondary interactions in solid state, presumably between the carbonyl groups of POM and the SBE-β-CD hydroxyl groups.

### 3.7 Dissolution study

**Figure 8:**
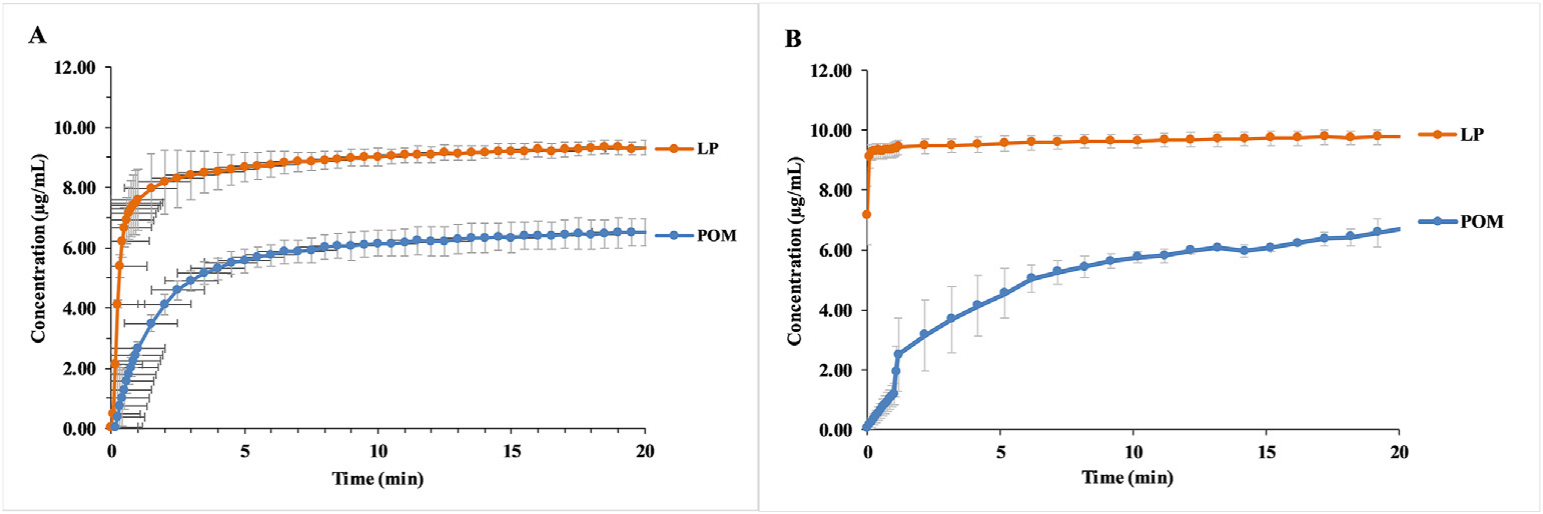
The dissolution profile of POM and LP at pH = 1.6 (A) and pH = 7 (B).

The dissolution profile of the examined POM and LP was followed in real-time via UV fiber-optic probes at two pHs. LP resulted in marked improvement of POM dissolution rate with respect to the drug alone regardless of pH. At pH 7, the dissolution kinetics were highly different, the lyophilized product reached the maximum concentration almost instantaneously, while the neat active ingredient needed 20 minutes to form a saturated solution (Figure 8). The equilibrium solubilities in these two cases were also different, the solution of the LP had almost 10 μg/mL concentration, which is more than 1.5 times higher, than the equilibrium concentration of the neat POM. At pH = 1.6 the difference between the LP and POM was not as conspicuous, as in the case of the measurement at pH = 7. But the LP reached the equilibrium concentration faster (1 min) than the POM (5 min) and also had a higher equilibrium solubility than the neat active ingredient (Figure 8A). It is also noteworthy that the intrinsic pharmacokinetic parameters of weakly and moderately bound guest molecules (K_s_<10000 M^−1^) is unaltered by CD complexation [35]. Thus, it can be assumed, that inclusion complex formation in case of POM-SBE-β-CD would not impact the pharmacokinetics of the active, only its solubility and implicitly its bioavailability.

**Figure 9:**
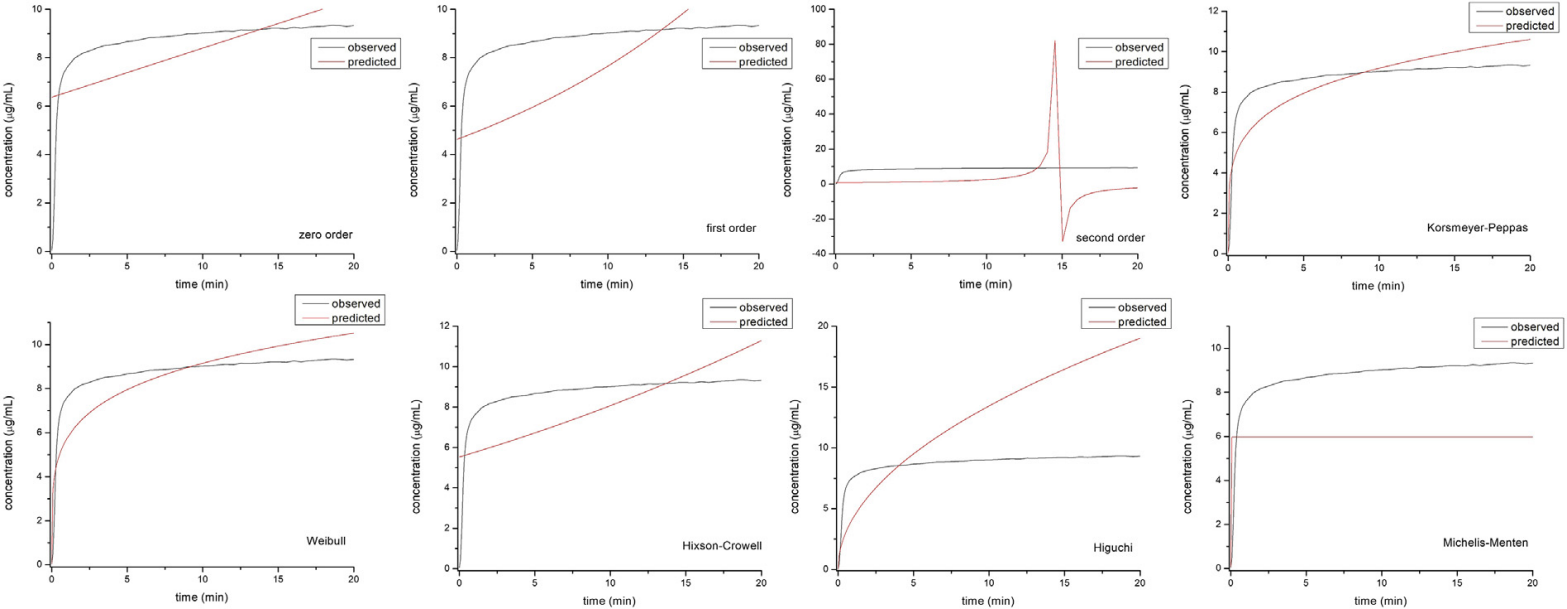
Kinetic models of POM release from LP at pH = 1.6.

**Figure 10:**
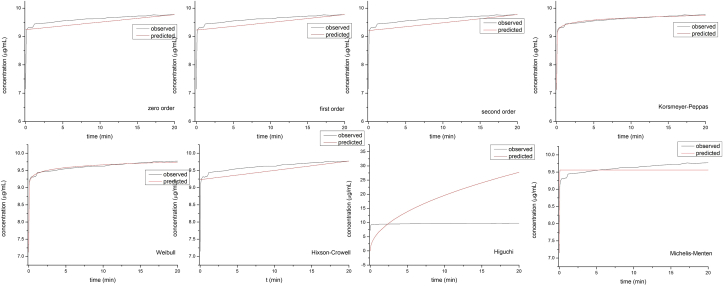
Kinetic models of POM release from LP at pH = 7.

To analyze the profile of drug release from LP, KinetDS 3.0 software was used [23]. The results of POM release at both pHs were fitted to eight different kinetic models as seen in Figures 9 and 10. The AIK, BIC, and RSME values were the best for describing the release kinetics of POM from the LP (Table 4 and Table 5). The kinetic models that best described the dissolution profiles were the Korsmeyer-Peppas and Weibull model. There is no significant difference between these two models and both were used previously to evaluate the kinetic release from inclusion complexes or from other matrix-type drug delivery system [24, 36].

**Table 4:** Results of data fitting for POM release from LP at pH = 1.

Model	Slope	Intercept	R^2^	R^2^_emp._	RMSE	AIC	BIC	*k*
Zero-order	0.2029	6.3709	0.3996	0.3996	1.6211	253.820	257.663	-
First-order	0.0502	1.5327	0.1534	-0.1382	2.2314	286.392	290.255	-
Second-order	-8.506	1.2473	0.0406	-47.38	14.552	477.652	481.516	-
Korsmeyer-Peppas	0.2092	1.7354	0.8507	0.6943	1.1567	219.371	223.234	5.67
Weibull	16.952	0.2115	0.8502	0.7128	1.1211	216.185	220.048	16.9
Hixson-Crowell	0.0237	1.7685	0.2390	0.2694	1.7882	263.806	267.670	0.02
Higuchi	0.5000	1.4479	-5.033	-5.033	5.1387	371.477	375.341	4.25
Michelis-Menten	1.9∗10^−9^	0.1676	0.9898	-0.8082	2.8133	310.027	313.890	1.2∗10^−8^

**Table 5:** Results of data fitting for POM release from LP at pH = 7.

Model	Slope	Intercept	R^2^	R^2^_emp._	RMSE	AIC	BIC	*k*
Zero-order	0.02650	9.2429	0.3492	0.3492	0.3396	70.260	73.735	-
First-order	0.00287	2.2222	0.3005	0.3454	0.3406	70.508	73.983	-
Second-order	0.00031	0.1085	0.2556	0.3387	0.3423	70.829	74.405	-
Korsmeyer-Peppas	0.01203	2.2406	0.9948	0.9936	0.0366	-123.92	-120.453	9.40
Weibull	0.01260	-2.3154	0.9946	0.9934	0.0341	-122.76	-119.291	10.13
Hixson-Crowell	0.00201	2.0979	0.3163	0.3469	0.3402	70.409	73.884	0.002
Higuchi	0.50000	1.8254	-873.9	-873.9	12.451	372.814	376.289	6.21
Michelis-Menten	3.5∗10^−12^	0.1046	0.8516	0.7655	0.2039	27.391	30.866	3.4∗10^−11^

### 3.8 *In vitro* antiproliferative assay

Some studies suggest that CD complexation can improve the pharmacodynamic properties of drugs, including anticancer agents as well [37, 38]. However, it should be noted that the use of CD is intended to increase the solubility of pharmaceuticals. The antiproliferative activity of POM, PM and LP was assessed by IC_50_ measured on human myeloma cell line LP-1, which has previously been shown to be adequate to study the antitumor activity of POM [39]. The determined IC_50_ value for POM (33.28μM ± 5.2), LP (37.28μM ± 7.3), and PM (39.29μM ± 8.5) indicating that complex formation did not significantly affect the anticancer effect of the thalidomide-analogue. However, it should be noted that thalidomide administered orally in combination with SBE-β-CD, led to a significant delay in tumor formation as a result of improved cellular drug absorption, distribution through solubilization in mice [13].

## 4 Conclusion

The physico-chemical profiling of POM was described for the first time and results underlined that the blockbuster drug is characterized by low solubility and low lipophilicity, which is independent of pH, which underlines the inclusion of POM as a BCS class IV drug. The present work also described the first method for solubility improvement of POM via CD complexation. Among the nine different CD derivatives tested, the most promising host molecule was revealed to be SBE-β-CD.

The binary system between POM and SBE-β-CD was fully characterized in liquid and solid state by state-of-art analytical techniques and molecular modelling. The inclusion complex formation resulted in significantly improved solubility, immediate release of the active without decreased antiproliferative activity. Using CD complex formation applying SBE-β-CD as host seems promising cheap, simple environmentally friendly tool to develop improved drug formulation containing POM.

## Declarations

### Author contribution statement

Zoltán-István Szabó: Conceived and designed the experiments; Wrote the paper.

György Orbán; Máté Dobó; Eszter Kiss; Lívia Budai; Judit Dobos: Performed the experiments.

Enikő Borbás; Szabina Kádár; Tamás Pálla: Performed the experiments; Analyzed and interpreted the data.

Dóra Csicsák: Conceived and designed the experiments; Performed the experiments.

Béla Fiser: Performed the experiments; Contributed reagents, materials, analysis tools or data.

Péter Horváth: Analyzed and interpreted the data.

László Őrfi: Analyzed and interpreted the data; Contributed reagents, materials, analysis tools or data.

Gergely Völgyi: Analyzed and interpreted the data; Wrote the paper.

Gergő Tóth: Conceived and designed the experiments; Analyzed and interpreted the data; Wrote the paper.

### Funding statement

Dr. Béla Fiser was supported by 10.13039/501100008530European Regional Development Fund (GINOP-2.3.4-15-2016-00004); This work was supported by the János Bolyai Research Scholarship of the 10.13039/501100003825Hungarian Academy of Sciences (G.T.) and Bolyai + New National Excellence Program (UNKP- 20- 5 - SE - 14) of the Ministry for Innovation and Technology is highly appreciated (G.T.).

### Data availability statement

Data will be made available on request.

### Declaration of interests statement

The authors declare no conflict of interest.

### Additional information

No additional information is available for this paper.

## References

[1] Ramasamy K., Gay F., Weisel K., Zweegman S., Mateos M.V., Richardson P. (2021). Improving outcomes for patients with relapsed multiple myeloma: challenges and considerations of current and emerging treatment options. Blood Rev..

[2] Dimopoulos M., Weisel K., Moreau P., Anderson L.D., White D., San-Miguel J., Sonneveld P., Engelhardt M., Jenner M., Corso A., Dürig J., Pavic M., Salomo M., Casal E., Srinivasan S., Yu X., Nguyen T.V., Biyukov T., Peluso T., Richardson P. (2020). Pomalidomide, Bortezomib, and Dexamethasone for Multiple Myeloma Previously Treated with Lenalidomide (OPTIMISMM): Outcomes by Prior Treatment at First Relapse, Leukemia.

[3] (2021). EMA assesment report. https://www.ema.europa.eu/en/documents/assessment-report/pomalidomide-celgene-epar-public-assessment-report_en.pdf.

[4] Kanaujia P., Poovizhi P., Ng W.K., Tan R.B.H. (2015). Amorphous formulations for dissolution and bioavailability enhancement of poorly soluble APIs. Powder Technol..

[5] Carrier R.L., Miller L.A., Ahmed I. (2007). The utility of cyclodextrins for enhancing oral bioavailability. J. Contr. Release.

[6] Jansook P., Ogawa N., Loftsson T. (2018). Cyclodextrins: structure, physicochemical properties and pharmaceutical applications. Int. J. Pharm..

[7] Brewster M.E., Loftsson T. (2002). The use of chemically modified cyclodextrins in the development of formulations for chemical delivery systems. Pharmazie.

[8] Szabó Z.I., Szőcs L., Muntean D.L., Noszál B., Tóth G. (2016). Chiral Separation of uncharged pomalidomide enantiomers using carboxymethyl-- cyclodetrin: a validated capillary electrophoretic method. Chirality.

[9] Szabó Z.I., Szőcs L., Horváth P., Komjáti B., Nagy J., Jánoska Á., Muntean D.L., Noszál B., Tóth G. (2016). Liquid chromatography with mass spectrometry enantioseparation of pomalidomide on cyclodextrin-bonded chiral stationary phases and the elucidation of the chiral recognition mechanisms by NMR spectroscopy and molecular modeling. J. Separ. Sci..

[10] Song J.X., Yan Y., Yao J., Chen J.M., Lu T.B. (2014). Improving the solubility of lenalidomide via cocrystals. Cryst. Growth Des..

[11] Kratz J.M., Teixeira M.R., Ferronato K., Teixeira H.F., Koester L.S., Simões C.M.O. (2012). Preparation, characterization, and in vitro intestinal permeability evaluation of thalidomide-hydroxypropyl-β-cyclodextrin complexes. AAPS PharmSciTech.

[12] Krenn M., Gamcsik M.P., Vogelsang G.B., Colvin O.M., Leong K.W. (1992). Improvements in solubility and stability of thalidomide upon complexation with hydroxypropyl-β-cyclodextrin. J. Pharmaceut. Sci..

[13] Kale R., Tayade P., Saraf M., Juvekar A. (2008). Molecular encapsulation of thalidomide with sulfobutyl ether-7 β-cyclodextrin for immediate release property: enhanced in vivo antitumor and antiangiogenesis efficacy in mice. Drug Dev. Ind. Pharm..

[14] Pálla T., Fogarasi E., Noszál B., Tóth G. (2019). Characterization of the site-specific acid-base equilibria of 3-nitrotyrosine. Chem. Biodivers..

[15] Tóth G., Mazák K., Hosztafi S., Kökösi J., Noszál B. (2013). Species-specific lipophilicity of thyroid hormones and their precursors in view of their membrane transport properties. J. Pharmaceut. Biomed. Anal..

[16] Connors K.A., Higuchi T. (1965). Phase solubility techniques. Adv. Anal. Chem. Instrum..

[17] Kiss E., Szabó V.A., Horváth P. (2019). Simple circular dichroism method for selection of the optimal cyclodextrin for drug complexation. J. Inclusion Phenom. Macrocycl. Chem..

[18] Orgován G., Kelemen H., Noszál B. (2016). Protonation and β-cyclodextrin complex formation equilibria of fluconazole. J. Inclusion Phenom. Macrocycl. Chem..

[19] Szabó Z.I., Ludmerczki R., Fiser B., Noszál B., Tóth G. (2019). Chiral separation of rasagiline using sulfobutylether-β-cyclodextrin: capillary electrophoresis, NMR and molecular modeling study. Electrophoresis.

[20] Jain A.S., Date A.A., Pissurlenkar R.R.S., Coutinho E.C., Nagarsenker M.S. (2011). Sulfobutyl ether 7 β-cyclodextrin (SBE 7 β-CD) carbamazepine complex: preparation, characterization, molecular modeling, and evaluation of in vivo anti-epileptic activity. AAPS PharmSciTech.

[21] Hetényi C., van der Spoel D. (2009). Efficient docking of peptides to proteins without prior knowledge of the binding site. Protein Sci..

[22] Trott O., Olson A.J., Vina AutoDock (2009). Improving the speed and accuracy of docking with a new scoring function, efficient optimization, and multithreading. J. Comput. Chem..

[23] Mendyk A., Jachowicz R., Fijorek K., Dorozyński P., Kulinowski P., Polak S., KinetDS (2012). An open source software for dissolution test data analysis. Dissolution Technol..

[24] Al-Qubaisi M.S., Rasedee A., Flaifel M.H., Eid E.E.M., Hussein-Al-Ali S., Alhassan F.H., Salih A.M., Hussein M.Z., Zainal Z., Sani D., Aljumaily A.H., Saeed M.I. (2019). Characterization of thymoquinone/hydroxypropyl-β-cyclodextrin inclusion complex: application to anti-allergy properties. Eur. J. Pharmaceut. Sci..

[25] Costa P., Sousa Lobo J.M. (2001). Modeling and comparison of dissolution profiles. Eur. J. Pharmaceut. Sci..

[26] Morival C., Oumari S., Lenglet A., Le Corre P. (2018). Clinical pharmacokinetics of oral drugs in the treatment of multiple myeloma. Hematol. Oncol..

[27] Szente L., Szemán J., Sohajda T. (2016). Analytical characterization of cyclodextrins: history, official methods and recommended new techniques. J. Pharmaceut. Biomed. Anal..

[28] Loftsson T. (2021). Cyclodextrins in parenteral formulations. J. Pharmaceut. Sci..

[29] Kelemen H., Noszal B., Orgovan G. (2020). Analysis of inclusion complexes of clotrimazole and differently substituted α, β and γ cyclodextrins by NMR spectroscopy. Rev. Chim. (Bucharest).

[30] Loftsson T., Hreinsdóttir D., Másson M. (2005). Evaluation of cyclodextrin solubilization of drugs. Int. J. Pharm..

[31] Medarević D., Kachrimanis K., Djurić Z., Ibrić S. (2015). Influence of hydrophilic polymers on the complexation of carbamazepine with hydroxypropyl-β-cyclodextrin. Eur. J. Pharmaceut. Sci..

[32] Connors K.A. (1997). The stability of cyclodextrin complexes in solution. Chem. Rev..

[33] Mura P. (2014). Analytical techniques for characterization of cyclodextrin complexes in aqueous solution: a review. J. Pharmaceut. Biomed. Anal..

[34] Das S.K., Kahali N., Bose A., Khanam J. (2018). Physicochemical characterization and in vitro dissolution performance of ibuprofen-Captisol® (sulfobutylether sodium salt of β-CD) inclusion complexes. J. Mol. Liq..

[35] Jones D.S., Dressman J.B., Loftsson T., Moya-Ortega M.D., Alvarez-Lorenzo C., Concheiro A. (2016). Pharmacokinetics of cyclodextrins and drugs after oral and parenteral administration of drug/cyclodextrin complexes. J. Pharm. Pharmacol..

[36] MacHín R., Isasi J.R., Vélaz I. (2012). β-Cyclodextrin hydrogels as potential drug delivery systems. Carbohydr. Polym..

[37] Gontijo S.M.L., Guimarães P.P.G., Viana C.T.R., Denadai Â.M.L., Gomes A.D.M., Campos P.P., Andrade S.P., Sinisterra R.D., Cortés M.E. (2015). Erlotinib/hydroxypropyl-β-cyclodextrin inclusion complex: characterization and *in vitro* and *in vivo* evaluation. J. Inclusion Phenom. Macrocycl. Chem..

[38] Oliveira A.P., Silva A.L.N., Viana L.G.F.C., Silva M.G., Lavor É.M., Oliveira-Júnior R.G., Alencar-Filho E.B., Lima R.S., Mendes R.L., Rolim L.A., Anjos D.S.C., Ferraz L.R.M., Rolim-Neto P.J., Silva M.F.S., Pessoa C. do Ó., Almeida J.R.G.S. (2019). β-Cyclodextrin complex improves the bioavailability and antitumor potential of cirsiliol, a flavone isolated from Leonotis nepetifolia (Lamiaceae). Heliyon.

[39] Verhelle D., Corral L.G., Wong K., Mueller J.H., De Parseval L.M., Jensen-Pergakes K., Schafer P.H., Chen R., Glezer E., Ferguson G.D., Lopez-Girona A., Muller G.W., Brady H.A., Chan K.W.H. (2007). Lenalidomide and CC-4047 inhibit the proliferation of malignant B cells while expanding normal CD34+ progenitor cells. Canc. Res..

